# The Role of Antibodies Against the Crude Capsular Extract in the Immune Response of Porcine Alveolar Macrophages to *In Vitro* Infection of Various Serovars of *Glaesserella* (*Haemophilus) parasuis*

**DOI:** 10.3389/fimmu.2021.635097

**Published:** 2021-04-22

**Authors:** Katarína Matiašková, Lenka Kavanová, Pavel Kulich, Jan Gebauer, Kateřina Nedbalcová, Hana Kudláčková, Radek Tesařík, Martin Faldyna

**Affiliations:** ^1^ Department of Infectious Diseases and Preventive Medicine, Veterinary Research Institute, Brno, Czechia; ^2^ Department of Pharmacology and Toxicology, Veterinary Research Institute, Brno, Czechia; ^3^ Department of Infectious Diseases and Microbiology, University of Veterinary Sciences, Brno, Czechia

**Keywords:** *Haemophilus parasuis*, *Glaesserela parasuis*, capsule, antibodies, porcine alveolar macrophages, reactive oxygen species, catalase, antioxidants

## Abstract

In Glässer’s disease outbreaks, *Glaesserella (Haemophilus) parasuis* has to overcome the non-specific immune system in the lower respiratory tract, the alveolar macrophages. Here we showed that porcine alveolar macrophages (PAMs) were able to recognize and phagocyte *G. parasuis* with strain-to-strain variability despite the presence of the capsule in virulent (serovar 1, 5, 12) as well in avirulent strains (serovar 6 and 9). The capsule, outer membrane proteins, virulence-associated autotransporters, cytolethal distending toxins and many other proteins have been identified as virulence factors of this bacterium. Therefore, we immunized pigs with the crude capsular extract (cCE) from the virulent *G. parasuis* CAPM 6475 strain (serovar 5) and evaluated the role of the anti-cCE/post-vaccinal IgG in the immune response of PAMs to *in vitro* infection with various *G. parasuis* strains. We demonstrated the specific binding of the antibodies to the cCE by Western-blotting assay and immunoprecipitation as well as the specific binding to the strain CAPM 6475 in transmission electron microscopy. In the cCE, we identified several virulence-associated proteins that were immunoreactive with IgG isolated from sera of immunized pigs. Opsonization of *G. parasuis* strains by post-vaccinal IgG led to enhanced phagocytosis of *G. parasuis* by PAMs at the first two hours of infection. Moreover, opsonization increased the oxidative burst and expression/production of both pro- and anti-inflammatory cytokines. The neutralizing effects of these antibodies on the antioxidant mechanisms of *G. parasuis* may lead to attenuation of its virulence and pathogenicity *in vivo*. Together with opsonization of bacteria by these antibodies, the host may eliminate *G. parasuis* in the infection site more efficiently. Based on these results, the crude capsular extract is a vaccine candidate with immunogenic properties.

## Introduction

*Glaesserella* (*Haemophilus) parasuis*, a gram-negative bacterium from the family *Pasteurellaceae*, is a commensal organism of the upper respiratory tract of swine (1). Under certain conditions, it is able to invade host and cause severe systemic disease (Glässer’s disease) with high morbidity and mortality (2). To date, 15 serovars of different virulence have been defined (3) but some differences in virulence have been observed also among strains of the same serovar indicating the presence of other virulence factors (4). In Glässer’s disease outbreaks, *G. parasuis* has to overcome the first line of defense in the lower respiratory tract of pigs, the porcine alveolar macrophages (PAMs) (2, 5). PAMs recognize the cell structures on the surface of the bacterium, phagocyte, lyse it and produce pro-inflammatory and anti-inflammatory cytokines and chemokines to attract the leucocytes to the infection site (6). Differences in phagocytosis observed in various *G. parasuis* strains may be caused by presence of the capsule, different structure of the capsule polysaccharides, phagocytosis resistance mechanism or other virulence factors like virulence associated autotransporters (VtaA) or transferrin binding protein B (TbpB) (4, 7–10).

Phagocytosis may be more effective when the bacterium is opsonized by a complement or by antibodies. Since it has been known that *G. parasuis* is resistant to serum complement (11) and bacterial capsule interferes with complement deposition (12), opsonization of the bacterium would be more effective by antibodies (7). Based on these facts, we decided to prepare the crude capsular extract (cCE) from the virulent *G. parasuis* strain that contains capsular polysaccharides and also proteins associated with the virulence of the bacterium (13) and thus antibodies against this antigens may opsonize the bacterium more efficiently. The protective role of antibodies against *G. parasuis* infection was proved in numerous *in vivo* experiments (14–16). In our previous study, the cCE was highly immunogenic and mice immunized with the cCE were partially protected against the challenge with various *G. parasuis* strains. Moreover, mice immunized with the cCE decreased bacterial load in the target tissues comparing to the non-immunized mice (13). The objective of the present study was to determine the role of antibodies against this capsular extract in the immune response of PAMs to *in vitro* infection with various *G. parasuis* strains opsonized by these antibodies. We hypothesized that these antibodies may facilitate phagocytosis of *G. parasuis* leading to higher production of reactive oxygen species (ROS) as well as to higher production of pro-inflammatory and anti-inflammatory cytokines with subsequent more effective destruction of the bacterium.

## Materials and Methods

### Bacterial Strains

The reference strains of *G. parasuis* kindly provided by Université de Montréal (Canada) used in this study were the following: strain No.4 of serovar (s.) 1 (HP1), strain 131 of s. 6 (HP6), strain D74 of s. 9 (HP9) and strain H425 of s. 12 (HP12). We used also the field strain CAPM 6475 of serovar 5 (HP5) which did originate from the brain of a pig with meningitis (14). Kielsten and Rapp-Gabrielson (3) determined the virulence of the serovar reference strains, whereby serovars 6 and 9 are non-virulent and serovars 1, 5 and 12 are virulent. Strains were grown on chocolate agar plates (LabmediaServis) at 37°C for 18 hours.

### Capsule Detection of *G. parasuis* Strains

Capsule was detected using the negative staining method. Each strain of *G. parasuis* used in the study was cultivated on chocolate agar overnight, resuspended in a phosphate-buffered saline (PBS, ThermoFisher Scientific), transported to the watch glasses and covered with a copper grid (300 OLD Mesh, Agar Scientific) coated with Formvar membrane (Sigma-Aldrich) and carbon (Agar Scientific). After 5 minutes, the grid was removed and residual water was dried out with a filter paper. Subsequently, a drop of 2% aqueous ruthenium red (Sigma-Aldrich) was added to the bacteria culture on the grid for a few seconds. After drying of stain residues with filtration paper, the sample was observed by the transmission electron microscope Philips 208 S MORGAGNI (FEI) at a magnification of 2,800 -18,000× and at accelerating voltage of 80 kV.

### Preparation Method of the Crude Capsular Extract

The preparation method of the crude capsular extract (cCE) obtained from G*. parasuis* strain CAPM 6475 by methanol extraction was described in our previous study (13). Briefly, the overnight culture of *G. parasuis* resuspended in D-PBS was incubated in a water bath at 60°C for 1 h and formaldehyde was added to the bacterial suspension to a final concentration of 0.2% (v/v). After centrifugation at 9,500 × g at 4°C for 1 h, the supernatant was concentrated to 1/50 of the volume using a VIVAFLOW 200 filter 5,000 MWCO PES (Sartorius Stedim Biotech) and three concentrate volumes of methanol (PENTA) and sodium acetate (PENTA) to 1% (w/v) were added. After 24 h, the supernatant was filtered with VIVAFLOW 200 filter 0.2 µm PES (Sartorius Stedim Biotech) and three concentrate volumes of acetone (PENTA) were added. After 36 h of the precipitate gravity settling, supernatant was discarded and the precipitate was resuspended in an aqua pro injection and stored at -20°C. The concentration of proteins in the cCE was determined with a Pierce™ BCA Protein Assay Kit (ThermoFisher Scientific) according to the manufacturer’s instructions. The concentration of polysaccharides was determined using Phenol-Sulfuric acid method (PSA) with glucose as a standard for calibration. The samples and glucose (PENTA) were dispensed into the 96-well plates and 75 µL of a sulfuric acid (PENTA) was added in each well. After 15 min of incubation at 90°C in the dark, 15 µL of 5% phenol water solution was added to each well. After 5 min of shaking, the absorbance was measured at 488 nm using a multireader Synergy H1 (BioTek).

### Antibodies Isolation

Sixteen pigs included in the experiment were negatively tested for presence of anti-G. parasuis antibodies before the immunization with the cCE by commercial ELISA kit (Swinecheck HPS ELISA test, Biovet). Animals were intramuscularly immunized with two 2 mL doses of the cCE and Montanide ISA 201 VG adjuvant (Seppic) in the ratio of 1:1 at 21-day intervals. The polysaccharide and protein concentration in one dose was 0.5 mg and 0.7 mg, respectively. Blood samples obtained from pigs before the immunization (negative sera) and at 14 days after the second immunization (positive sera) were centrifuged at 830 × g at 21°C for 15 min, sera were collected, mixed together (negative or positive pooled serum) and inactivated at 56°C for 30 min. The IgG antibodies were then purified from the negative or positive pooled serum using the column with Protein A (Protein A IgG Purification Kit, Thermo Scientific) according to the manufacturer’s instructions. For *in vitro* tests, the concentration of IgG isolated from the positive pooled serum (post-vaccinal/anti-cCE IgG) was measured with a Pierce™ BCA Protein Assay Kit. All procedures involving animals were approved by the Branch Commission for Animal Welfare of the Ministry of Agriculture of the Czech Republic (approval protocol No. MZe 1704).

### Detection of the Antibodies Against the cCE

The antibody titer against the cCE in the negative or the positive sera of pigs was determined by in house indirect enzyme-linked immunosorbent assay (ELISA) (13). The cCE antigen was dissolved in a carbonate-bicarbonate buffer (pH 9.6) in a concentration of 1 μg/mL that was subsequently dispensed (100 μL/well) into the wells of the Nunc MaxiSorp^®^ flat-bottom 96-well polystyrene microtiter plate (ThermoFisher Scientific). After the overnight incubation at 4°C, the wells were rinsed four times with 300 μL of diluting solution containing PBS with 0.05% Tween-20 (PBS-T). The wells were then blocked with 0.5% casein and 10% sucrose (in 250 μL of PBS per well) for 30 min at a room temperature (RT). The tested sera were prediluted 300× in a solution of PBS with 0.05% Tween and 0.5% casein hydrolysate and dispensed to wells (100 μL per well). After the incubation of the plate for one hour at RT, the wells were rinsed four times with PBS-T. 100 μL of goat anti-porcine IgG labelled with horseradish peroxidase (Bethyl Laboratories) prediluted 1:30 000 in PBS with 0.05% Tween and 0.5% casein hydrolysate was added to wells and incubated for another 1 h at RT. Afterwards, the plate was rinsed four times with PBS-T and 100 μL of TMB-Complete 2 solution (TestLine) was added into each well. The reaction was stopped after 15 min of incubation at RT by adding 50 μL of 1M sulfuric acid into each well. The absorbance was read at 450 nm using the multi-detection microplate reader Synergy H1 (BioTek).

### The Specificity of the Antibodies Against the cCE

To prove the specificity of the antibodies against the cCE, Western-blotting, immunoprecipitation with mass spectrometry and transmission electron microscopy (TEM) were performed.

In the Western-blotting, the cCE antigen was resolved on 12.5% SDS-PAGE and blotted on PVDF membrane (Amersham). The membrane was then incubated overnight at 4°C in blocking solution containing 1% casein in PBS with 0.05% Tween-20 (PBS-T, Serva). The membranes were incubated with the positive or negative pooled serum or with the isolated IgG from these sera (diluted 100 × in PBS-T with 1% casein) for 1 h at RT. The membranes were then washed in PBS-T and incubated with goat anti-pig HRP-conjugated IgG (Bethyl Laboratories) for another 1 h at RT. Secondary antibodies were diluted 1:1000 in PBS-T. Specific protein bands were visualized with 3, 3´-diaminobenzidine (Sigma-Aldrich).

Immunoprecipitation protocol was established using Pierce Crosslink Immunoprecipitation Kit (Thermo scientific) according to manufacturer’s manual. Briefly, 50 µg of isolated IgG from positive or negative sera were bound to Protein A/G on agarose resin. After washing steps, bound antibodies were crosslinked with DSS (disuccinimidyl suberate) dissolved in DMSO to make a covalent bond. 2 mg of cCE was pre-cleared using the control agarose resin to prevent unspecific binding to agarose itself. Immunoprecipitation of 1 mg of pre-cleared cCE with isolated IgG took place at 4°C overnight with gentle mixing. After washing steps, specifically bound bacterial proteins were eluted with low pH elution buffer and analyzed by mass spectrometry.

### Immunoelectron Microscopy

Immunoelectron microscopy was performed according to Huebner et al. (17). The overnight culture of *G. parasuis* CAPM 6475 cultivated on the chocolate agar was resolved in 1 mL of PBS and centrifuged at 4,000 × g for 3 min. The bacterial pellet was then washed three times and resuspended in PBS. Drops (30 μL) of the bacterial culture were placed on Parafilm and a Formvar coated nickel grids (300 OLD Mesh, Agar Scientific) were placed on each drop for 1 min. The grids were then blocked by placement on drops (20 μL) of 0.5% fishskin-gelatin in PBS containing 0.1% Tween-20 (Serva) for 5 min. The grids were subsequently incubated on a drop (20 μL) of 1:50 diluted positive or negative pooled serum. After 20 min of incubation at RT, the grids were washed twice with PBS-T and placed on drops (20 μL) of 1:10 diluted Protein A-20 nm colloidal gold (Sigma-Aldrich) for 20 min at RT. Afterwards, the grids were washed four times with distilled water and observed by transmission electron microscope Philips 208S MORGAGNI (FEI) at a magnification of 7,500 × and at accelerating voltage of 80 kV.

### LC-MS/MS Analysis of the cCE and Immunocomplexes

By mass spectrometry, the protein content in the cCE and proteins of cCE bounded to the isolated IgG were analyzed. Fifty micrograms of proteins for each of four cCE replicates (four cCE preparations) were analyzed. For identification of proteins after immunoprecipitation, the whole elution fraction (without protein concentration determination) was used. All samples were prepared using FASP (filter-aided sample preparation) method (18). Every sample was washed 6 times in 8 M urea (Serva) in Vivacon 500 centrifugal tube (Sartorius Stedim) with 10,000 MWCO membrane filter. Dithiothreitol (10 mM, Sigma-Aldrich) and iodoacetamide (50 mM, Serva) in 25 mM TEAB buffer (triethylammonium bicarbonate, Sigma-Aldrich) were used for reduction and alkylation, respectively. Proteins were then digested with trypsin (Promega) in 1:50 ratio, for one hour at 37°C and then overnight at 25°C. After centrifugation, the eluate with digested peptides was evaporated (DNA120 SpeedVac, Thermo Savant, USA), peptide pellet was resuspended in 0.1% aqueous formic acid (Sigma- Aldrich) which serves as a mobile phase for liquid chromatography (UltiMate 3000 RSLCnano, Thermo Scientific). For separation and elution of peptides, 2-hour gradient with increasing concentration of acetonitrile (0.1% formic acid in acetonitrile, Sigma-Aldrich) at a flow rate of 300 nl/min was used. Peptides were separated on a 15 cm column (Acclaim PepMap RSLC C18, 2 µm, 100 A, 75 µm I.D., Thermo Scientific). uHPLC was connected to EASY-spray ion source and Orbitrap Velos Pro mass spectrometer (Thermo Scientific). A survey scan over m/z range 390-1,700 was used to identify protonated peptides with charge states of at least 2, which were automatically selected for data- dependent MS/MS analysis and fragmented by collision with helium gas. Ten fragment mass spectra after each full scan were recorded. Measured spectra were then searched using Proteome Discoverer (version 1.4, Thermo Scientific) with Sequest HT as a searching algorithm. Oxidation of M, deamidation of N and Q as a dynamic, and carbamidomethylation of C as a static modification was used. Precursor and fragment mass tolerances were set up as 10 ppm and 0.5 Da, respectively. Uniprot database for *Glaesserella* strain (from 2016/02) was used in Sequest HT. Only peptides with a false discovery rate less than 0.01 were considered as well identified and only proteins with at least two unique well identified peptides were included into results. Quantity of identified proteins is expressed by the PSM number (peptide spectrum matches). The ratio between vaccinated and control sample is calculated from the area under the curve of the chromatographic peak detected by mass spectrometer.

### Porcine Alveolar Macrophages Preparation

Porcine alveolar macrophages were obtained immediately after euthanasia of six clinically healthy 8-weeks-old pigs free of anti-*G. parasuis* antibodies (Swinecheck HPS ELISA test, Biovet) by bronchoalveolar lavage as previously described (19). Euthanasia was performed by intravenous injection of the anesthetic T61 (Intervet International B.V.) in a dose recommended by the manufacturer (5 mL/kg of body weight). All procedures involving animals were approved by the Branch Commission for Animal Welfare of the Ministry of Agriculture of the Czech Republic (approval protocol No. MZe 1487). After lavage, PAMs were washed three times with a calcium-magnesium free Dulbecco’s phosphate-buffered saline (D-PBS; Lonza) and aliquots with isolated PAMs were frozen in medium containing 75% RPMI-1640 (Sigma-Aldrich), 20% fetal bovine serum (FBS; PAA Laboratories), and 5% dimethyl sulfoxide (DMSO; Sigma-Aldrich) and stored in a liquid nitrogen until use. Before each experiment, PAMs were thawed in a water bath at 37°C. The cell viability after freezing and thawing was determined by the BD FACSAriaTM Fusion flow cytometer and was greater than 90%. The cells were washed with Dulbecco’s Modified Eagle’s Medium (DMEM, Invitrogen), centrifuged at 1,100 × g and 10°C for 10 min and resuspended with DMEM supplemented with 10% FBS and 1% antibiotics (Antibiotic Antimycotic Solution 100 ×: 10,000 units penicillin, 10 mg streptomycin, and 25 μg amphotericin B per mL; Sigma-Aldrich). PAMs were then dispensed into 24-well culture plates (Biotech) at a concentration of 4 × 10^5^ cells in 1 mL per well or in case of chemiluminescence assay into Nunc-Immuno™ MicroWell™ 96-well polystyrene plates (Sigma-Aldrich) at a concentration of 1 × 10^5^ cells in 0.2 mL per well and incubated overnight at 37°C, 5% CO_2_.

### Infection of PAMs

After overnight incubation at 37°C on chocolate agar plates, bacteria were harvested by centrifugation at 4,000 × g for 3 min following resuspending in 1 mL of D-PBS, washed twice with D-PBS and resuspended in D-PBS by adjusting turbidity to the density equivalent to a 2.5 McFarland standard (DENSI-LA-METER II, ErbaLachema). To determine the final bacterial concentration after washing, ten-fold dilutions were plated on chocolate agars. The concentration of the final inoculum was 1 × 10^9^ CFU/mL.

Before the infection, 4 × 10^6^ CFU of every bacterial strain was incubated with 180 μg of the isolated post-vaccinal IgG or without IgG for 30 min at 37°C.

After overnight incubation of PAMs in 24-well plates (37°C, 5% CO_2_), the non-adherent PAMs were washed with DMEM supplemented with 10% FBS. Thereafter, duplicate wells with PAMs were inoculated with *G. parasuis* strains pre-incubated with/without the isolated post-vaccinal IgG at a multiplicity of infection (MOI) of 10 in DMEM supplemented with 10% FBS. The infected and non-infected (only with DMEM) cells incubated at 37°C and 5% CO_2_ for different time points were then used in various assays (described in the chapters below).

### Phagocytosis Assay and Survival of Bacteria

Bacteria pre-incubated with/without the post-vaccinal IgG were allowed to uptake by PAMs for 0.5, 1, 2 or 5 h. After incubation, wells were washed twice and incubated with DMEM supplemented with 10% FBS and 5 mg/mL of gentamicin to kill the extracellular bacteria for additional 30 min at 37°C, 5% CO_2._ This concentration of gentamicin did not affect PAMs and was bactericidal to all strains. After 30 min with gentamicin, wells were washed twice with D-PBS and PAMs were lysed with 1% Saponin (Sigma-Aldrich). Bacteria were counted by plating ten-fold dilutions on chocolate agar plates.

To find out whether internalized bacteria survive inside the macrophages, PAMs were incubated with opsonized or non-opsonized bacteria for 2 h, washed twice with DMEM with 10% FBS and then gentamicin (5 mg/mL) was added to eliminate the extracellular bacteria. After 30 min at 37°C and 5% CO_2_, cells were incubated in DMEM with 10% FBS (without gentamicin) for additional 2, 4, 8 or 24 h. At these time points, cells were washed twice with D-PBS, lysed with 1% Saponin and survived bacteria were counted by plating.

### ROS Production

Production of reactive oxygen species (ROS) by *in vitro* infected PAMs was measured using chemiluminescence (CL) assay (20). After overnight incubation of PAMs in 96-well plates (37°C, 5% CO_2_), the wells were washed with Hanks’ balanced salt solution (HBSS, Lonza) and luminol-derivative L-012 (Wako Chemicals GmbH) was added to amplify the CL induced by respiratory burst of stimulated PAMs. L-012 was diluted in HBSS to the final concentration 0.15 mmol/L. A suspension of *G. parasuis* (MOI 10) pre-incubated with/without the post-vaccinal IgG was then added to the wells containing luminol L-012. The same volume of HBSS was added to the non-infected PAMs that served as a control. Chemiluminescence was measured at 37°C using a multi-detection microplate reader Synergy H1 (BioTek) in kinetic mode for 1 h. The results are expressed as integrals of chemiluminescence intensity (per 1 × 10^5^ viable cells) induced in PAMs with infection, and data are presented as percentage relative to the non-infected control.

### Quantitative RT-PCR

The total RNA was isolated from the infected and the non-infected PAMs at 4 and 24 h post infection (PI) by using RLT buffer (Qiagen) and an RNeasy Mini Kit (Qiagen) following manufacturer’s instructions and then reverse transcribed with oligo-dT primer and M-MLV reverse transcriptase (Invitrogen). Primers for IL-1RN gene were designed using the freely available Basic Local Alignment Search Tool (http://www.ncbi.nlm.nih.gov/blast/Blast.cgi). The remaining primers were designed according to the published papers and all are listed in **Table 1**. For real time PCR, LightCycler 480 II instrument (Roche) with a 384-well plate block was used. Each PCR reaction consisted of QuantiTect SYBR Green master mix (Qiagen), 1 μM of each primer and 0.5 μL of cDNA in a total volume of 3 μL. Briefly, PCR assay was performed under the following conditions: 95°C for 15 min; 45 x 95°C for 15 s, 58°C for 30 s, 72°C for 30 s. Melting analysis was performed at 60-95°C. Samples from each well were run in triplicate. Genes expression were calculated as multiples of the reference gene expression (Hypoxanthine phosphoribosyltransferase, HPRT) using the following formula: (1/(2^Ctgene^))/(1/(2^CtHPRT^)) (24). The final value of the expression is shown as the ratio of gene expression in the infected sample to expression in the non-infected.

**Table 1:** Primers used for the real-time PCR quantification of IL-1ß, TNF−α, IL-10, IL-1RN and HPRT.

Target gene	Primers	Name/Function/Reference
IL−1ß	*F:* GGGACTTGAAGAGAGAAGTGG	Interleukin 1 beta
	*R:* CTTTCCCTTGATCCCTAAGGT	Pro-inflammatory cytokine/ (21)
TNF−α	*F:* CCCCCAGAAGGAAGAGTTTC	Tumor necrosis factor
	*R:* CGGGCTTATCTGAGGTTTGA	Pro-inflammatory factor/ (22)
IL−10	*F:* TGAAGAGTGCCTTTAGCAAGCTC	Interleukin 10
	*R:* CTCATCTTCATCGTCATGTAGGC	Anti-inflammatory cytokine/ (23)
IL-1RN	*F:* AGGGAAGCTGTGCCTGTCCTGTG	Interleukin-1 receptor antagonist
	*R:* GGCCACTGTCGGAGCGGATGAAG	Antagonist of receptor for cytokine IL-1 beta/In this study
HPRT	*F:* GAGCTACTGTAATGACCAGTCAACG	Hypoxanthine phosphoribosyltransferase
	*R:* CCAGTGTCAATTATATCTTCAACAATCAA	Housekeeping gene/ (19)

### Cytokine Assay

Production of IL-1ß, TNF-α, IL-8, IL-10 and IL-1Ra at a protein level was measured in the cell culture supernatants from PAMs infected with *G. parasuis* for 4 and 24 h by commercial ELISA kits (R&D systems) according to the manufacturer’s instructions.

### Statistical Analysis

Data were analyzed using the Wilcoxon matched-pair singed rank test in GraphPad Prism (GraphPad Software, Inc.). A P-value less than 0.05 was considered significant. In graphs, data are expressed as means ± SEM.

## Results

### The Capsule Detection

**Figure 1:**
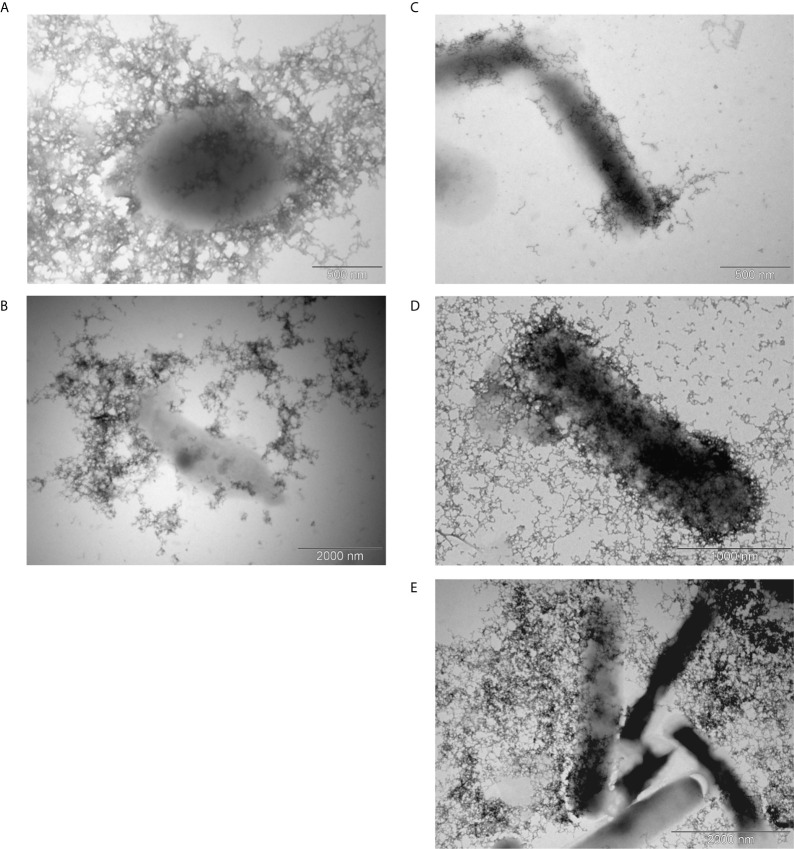
Transmission electron microscopy of the capsule. Encapsulated *G. parasuis* avirulent strains **(A, B)** and virulent strains **(C–E)** are following: strain 131 of serovar 6 **(A)**, strain D74 of serovar 9 **(B)**, strain No.4 of serovar 1 **(C)**, strain CAPM 6475 of serovar 5 **(D)**, and strain H425 of s. 12 **(E)**.

Using the negative staining and transmission electron microscope, we observed the capsule in all *G. parasuis* strains (**Figure 1**).

### Antibodies Against the Crude Capsular Extract

**Figure 2:**
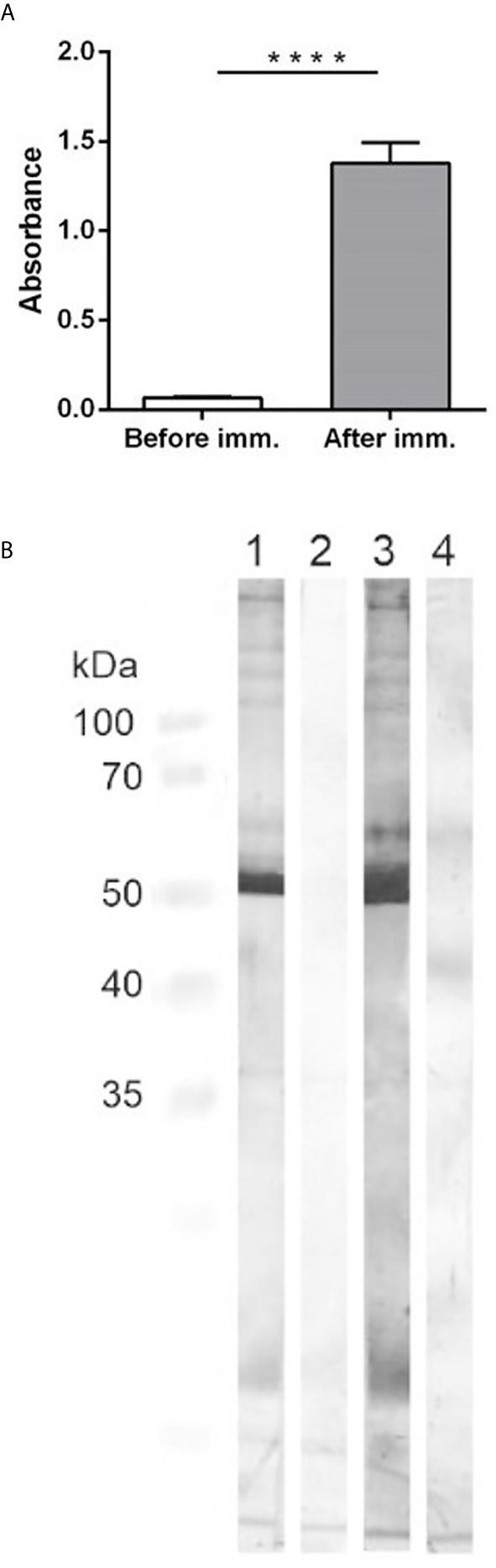
**(A)**: ELISA detection of specific antibodies against the crude capsular extract. The graph shows the antibody titer of antibodies in sera from pigs before the immunization and at 14 days after the second immunization with the cCE. Significant difference between the groups is denoted by asterisks (p<0.0001). **(B)**: Western-blotting. Antigen – the crude capsular extract. Primary antibodies: 1- IgG isolated from the positive pooled serum, 2 - IgG isolated from the negative pooled serum, 3 – positive pooled serum from pigs after the second immunization, 4 – negative pooled serum from pigs before the immunization. Secondary antibodies: goat anti-pig HRP-conjugated IgG. Specific protein bands were visualized with 3, 3´-diaminobenzidine.

Immunization of pigs with the crude capsular extract led to humoral immune response that was confirmed by the in-house indirect ELISA. Whereas, the anti-cCE IgG antibodies were not detected before the immunization, after the second immunization with the cCE the anti-cCE IgG were observed in significantly high levels (p<0.0001) (**Figure 2A**).

The Western-blotting showed that the antibodies from the immunized animals reacted with proteins in the cCE of different molecular weights, which was not observed in case of the antibodies from the non-immunized pigs (**Figure 2B**). The strongest band on the membrane was in the area about 50 kDa. Due to the preparation method of the cCE, we expected protein content and thus we analyzed it by mass spectrometry. The crude capsular extract contained more than 100 bacterial proteins (**Supplementary Material 1**), like outer membrane proteins (Omp P5, Omp P2), virulence-associated autotransporters (VtaA) or cytolethal distending toxins (CDT). The most abundant protein found in the extract was Catalase (Uniprot accession number U4RLR7) with a molecular weight of 54.9 kDa.

**Figure 3:**
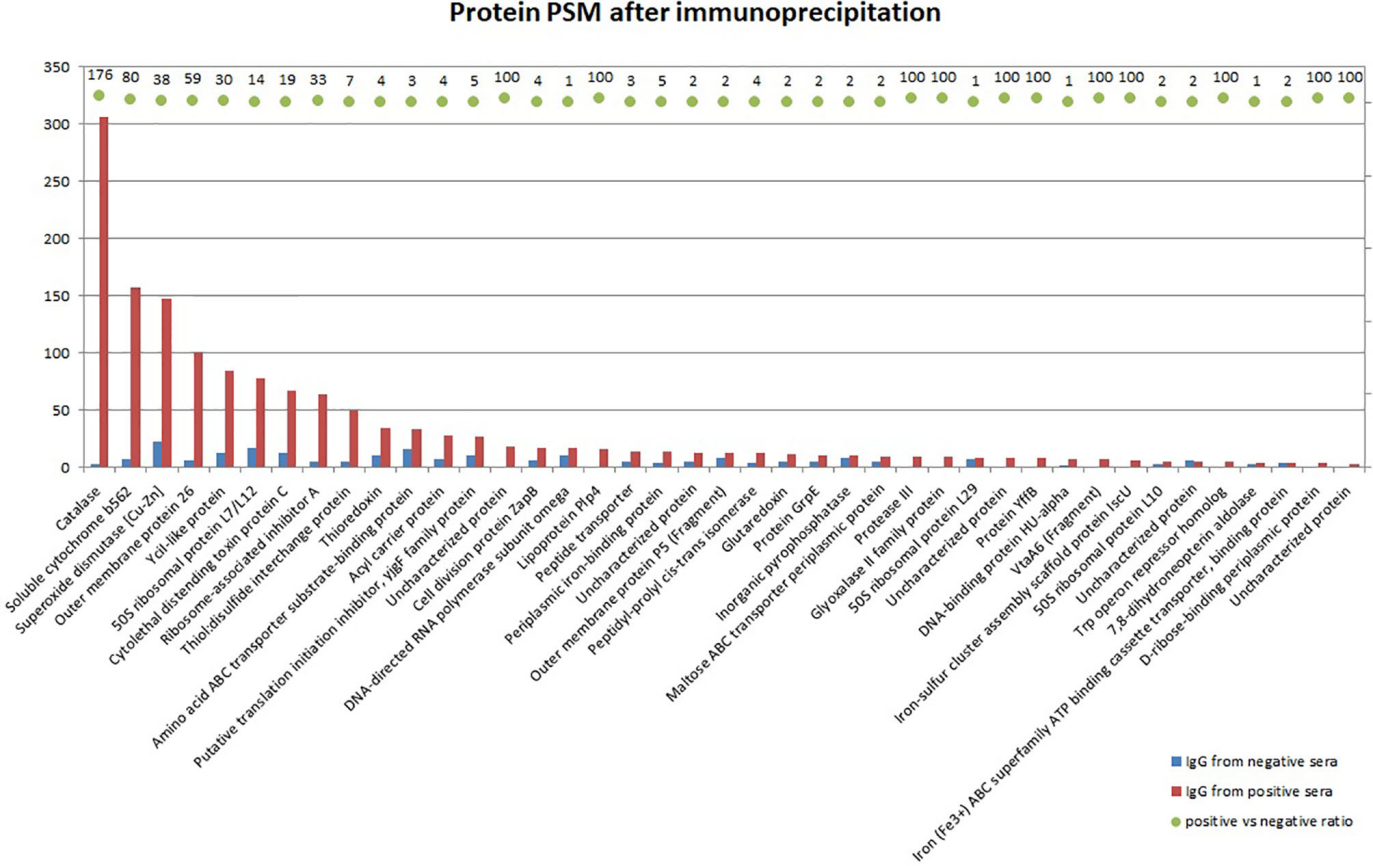
Mass spectrometry analysis of bacterial proteins from the crude capsular extract precipitated with IgG antibodies isolated from positive or negative pooled serum. Bars in the graph show identified protein quantity by PSM (peptide spectrum match), whereas the ratio between positive and negative sera is expressed by AUC (area under the curve) for each corresponding protein. The ratio which equals 100 is maximum defined value meaning that the protein is not detected in negative sera at all.

To determine immunogenic proteins in the capsular extract, we performed the immunoprecipitation of the capsular extract with IgG isolated from positive or negative pooled serum and analyzed captured bacterial proteins by mass spectrometry. The most reactive proteins with positive serum previously described as virulence factors in *G. parasuis* were catalase, superoxide dismutase, Omp 26, CdtC, Plp4, Omp 5, and VtaA6 (**Figure 3**).

**Figure 4:**
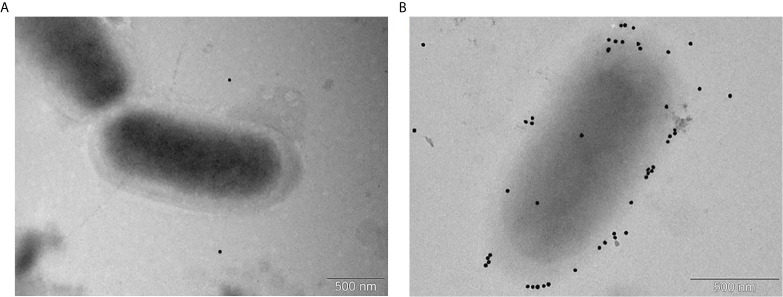
Immunoelectron microscopy of *G. parasuis* strain CAPM 6475 incubated with pooled sera obtained from pigs before the immunization **(A)** or after the second immunization with the cCE **(B)**. Serum antibodies bound to bacteria are indicated by gold particles (black spots) bound to Protein A.

To confirm and visualize binding of the post-vaccinal antibodies with *G. parasuis*, we performed the transmission electron microscopy of *G. parasuis* strain CAPM 6475 incubated with positive or negative sera using the protein A conjugated with 20nm colloidal gold. As we expected, we observed bacteria with bound antibodies visualized with gold particles only in a case of incubation with positive pooled serum (**Figure 4**).

### Phagocytosis Assay and Survival Assay After Phagocytosis

**Figure 5:**
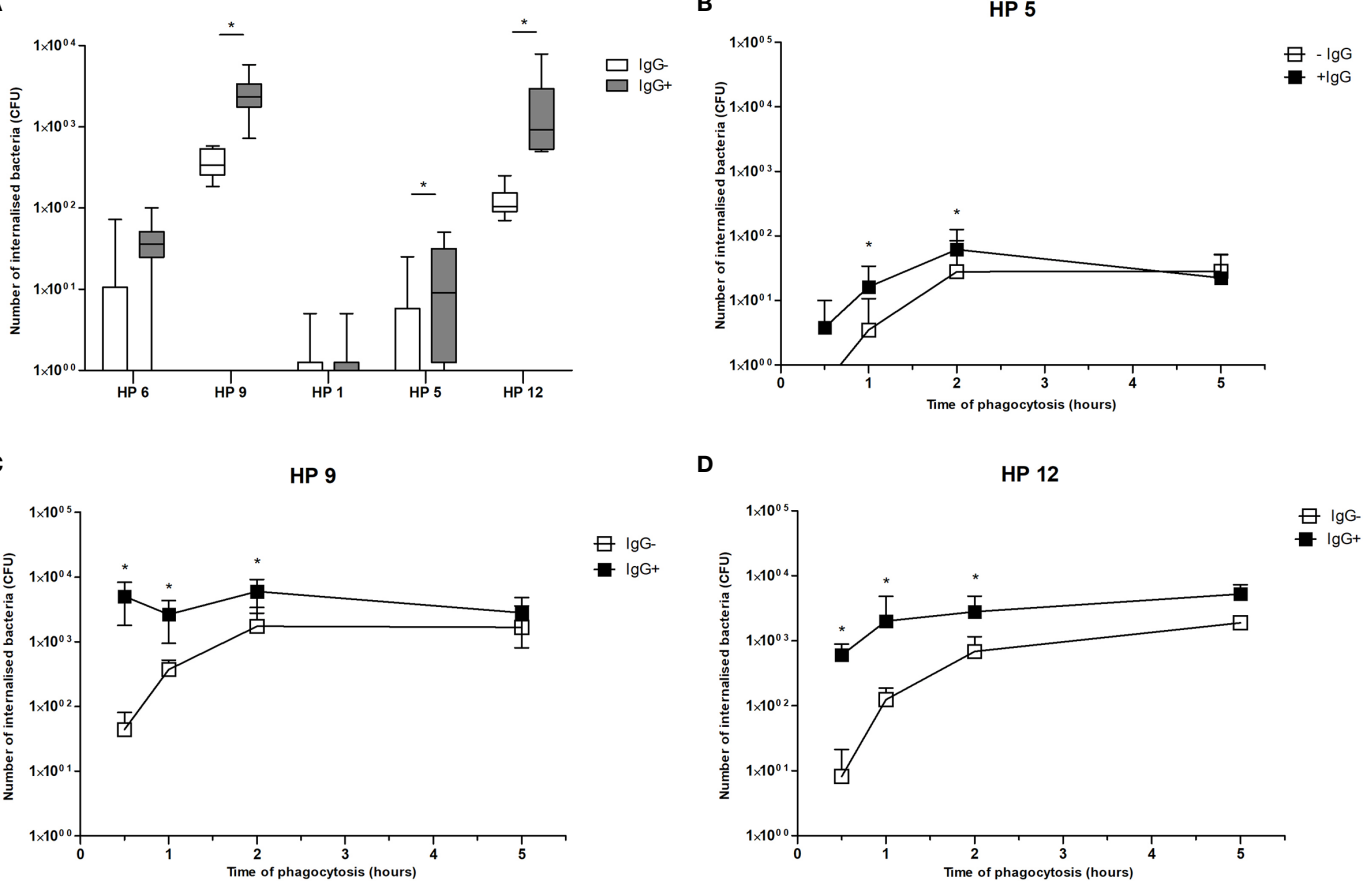
Phagocytosis assay. Data show presence of *G. parasuis* strains pre-incubated with (IgG+) or without (IgG-) the post-vaccinal IgG in lysed PAMs after one hour of phagocytosis **(A)** or after 0.5, 1, 2 and 5 h of phagocytosis **(B–D)**. Significant difference between groups with or without the post-vaccinal IgG is denoted by asterisk (p<0.05).

Survival of each *G. parasuis* strain inside PAMs 1 h after phagocytosis is shown in **Figure 5A**. Bacteria of serovar 9, 5 and 12 pre-incubated with the post-vaccinal IgG were present inside PAMs in significantly higher amounts than bacteria pre-incubated without the antibodies. On the other hand, opsonization of serovar 1 and 6 had no effect on their phagocytosis. To see kinetics of internalization process of bacteria, we performed phagocytosis assay in different time points. We chose bacterial strains (i.e. of serovar 9, 5, and 12) with a significantly higher number of ingested bacteria after opsonization compared to non-opsonized, which was observed after one hour of phagocytosis. This experiment revealed that opsonization of bacteria led to obviously higher uptake of bacteria up to 2 h of phagocytosis (**Figures 5B–D**). At 5 h PI, there was no difference in phagocytosis of the opsonized and non-opsonized bacteria.

In addition, to find out whether internalized bacteria survive inside PAMs, we determined number of viable bacteria in different time points after their internalization. Results of this experiment revealed that survival of ingested bacteria was not influenced by presence of IgG. Number of internalized bacteria of serovar 9 pre-incubated with IgG and without IgG was reduced to 69.05% and 33.33% in 2 h, to 11.59% and 1.67% in 4 h and to 0.28% and 0.00% in 8 h, respectively. Number of internalized bacteria of serovar 12 pre-incubated with IgG and without IgG was reduced to 63.19% and 56.08% in 2 h, to 28.87% and 22.85% in 4 h and to 4.60% and 12.58% in 8 h, respectively. Bacteria of both serovars were killed in 24 h. In addition, bacteria of serovar 5 were killed in 2 h after phagocytosis.

### ROS Production

**Figure 6:**
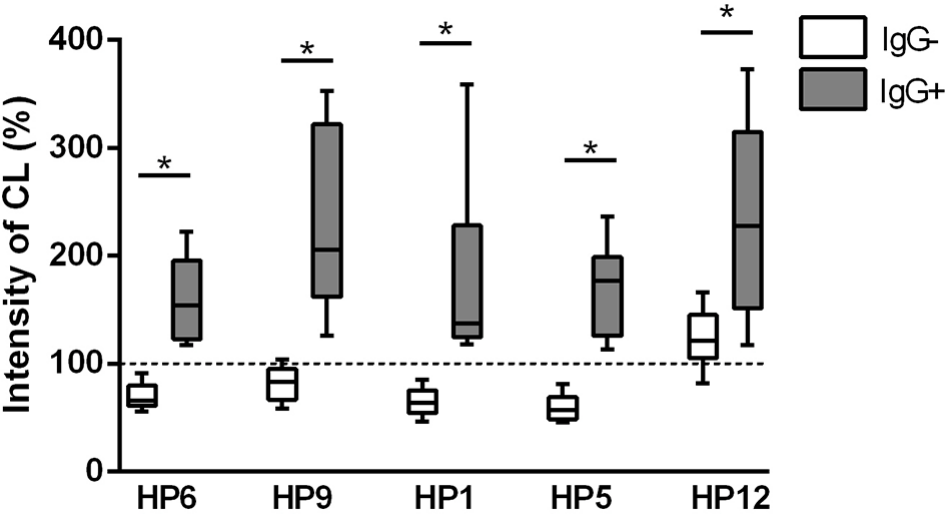
ROS production. ROS production was measured for 1 h by chemiluminescence assay in PAMs infected with various strains of *G. parasuis* pre-incubated with (IgG+) or without the post-vaccinal IgG (IgG-). Data are presented as percentage of integral of the chemiluminescence signal levels compared to the non-infected PAMs. Significant difference between groups with or without the IgG is denoted by asterisk (p<0.05).

Respiratory burst was measured using chemiluminescence assay in this study. Results of ROS production by PAMs during one hour of infection are expressed as integrals of the chemiluminescence signal and are shown in the **Figure 6**. All *G. parasuis* strains (except for serovar 12) without post-vaccinal IgG reduced ROS production below the level of spontaneous ROS production in the non-infected cells. On the other hand, opsonization of every *G. parasuis* strain with the post-vaccinal antibodies led to higher intensity of the CL signal in the infected PAMs comparing to PAMs affected with the non-opsonized bacteria.

### The Immune Response of PAMs to *In Vitro* Infection

**Figure 7:**
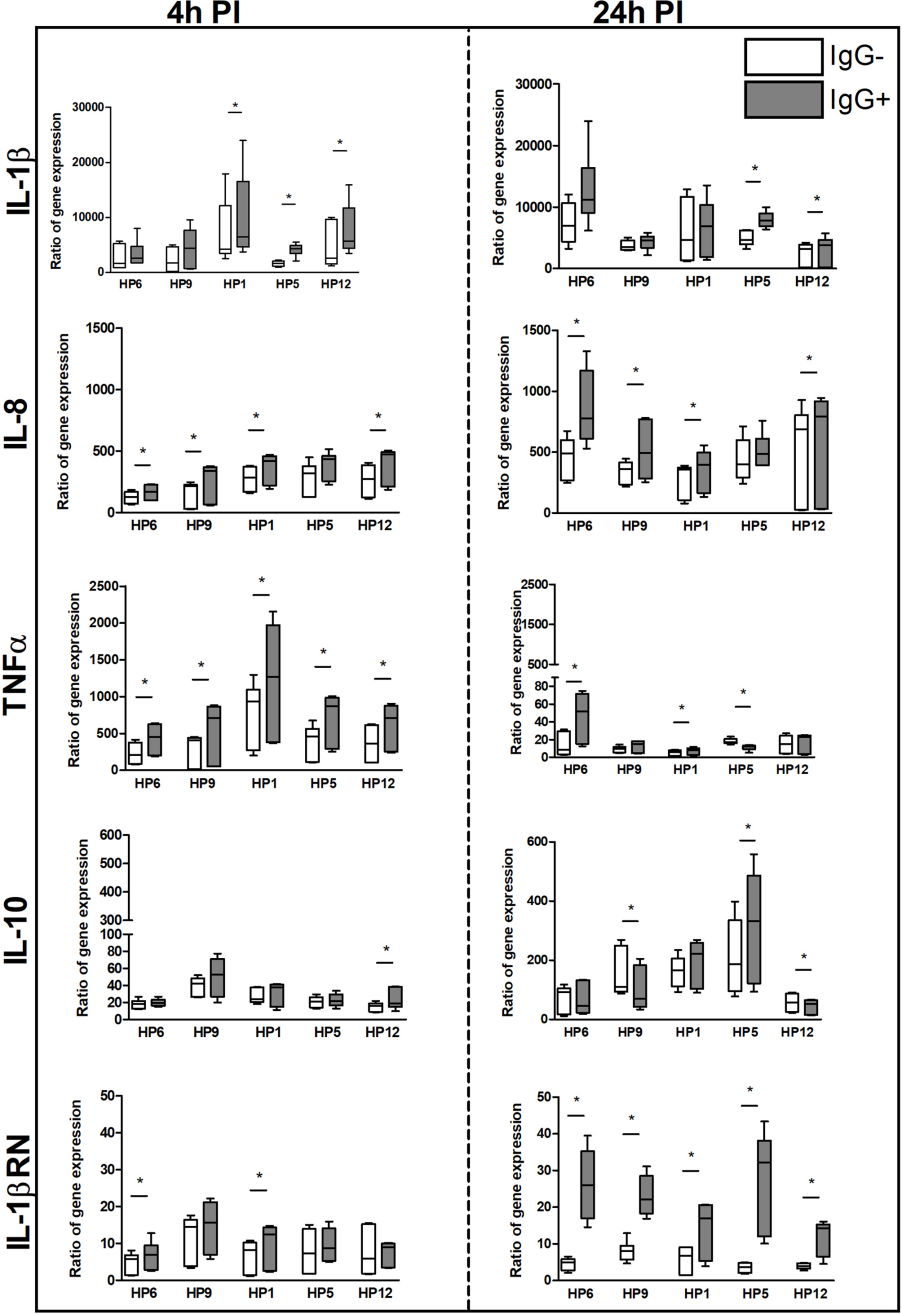
The immune response of PAMs at the transcriptional level. PAMs were infected with various *G. parasuis* strains for 4 and 24 h. The mRNA expression of IL-1β, IL-8, TNF-α, IL-10 and IL-1RN was measured by real-time PCR at these PI times. The degree of gene expression was calculated as a fold of the reference gene expression, hypoxanthine phosphoribosyltransferase, and final results are displayed as the ratio of gene expression in the infected sample to expression in the non-infected. Significant difference between groups with (IgG+) or without (IgG-) the post-vaccinal IgG is denoted by asterisk (p<0.05).

Results of mRNA expression of IL-1β, IL-8, TNF-α, IL-10 and IL-1RN gene at 4 and 24 h PI are shown in **Figure 7**. Higher mRNA expression of pro-inflammatory TNF-α and anti-inflammatory IL-1RN gene was detected in PAMs infected with every *G. parasuis* strain pre-incubated with post-vaccinal IgG than without IgG at 4 h PI and 24 h PI, respectively. This phenomenon was observed also in case of IL-8 when PAMs were infected for 4 h or 24 h with opsonized bacterial strains, except for serovar 5. Expression of IL-1β was higher in PAMs affected with virulent serovars for 4 h and with serovar 5 or 12 also for 24 h in the presence of IgG than without IgG. IL-10 was up-regulated in PAMs incubated with serovar 12 for 4 h and with serovar 5 for 24 h in the presence of IgG.

IL-8, IL-10 and IL-RN genes were detected up-regulated more at 24 h PI than at 4 h PI in all groups at the transcriptional level. On the contrary, levels of mRNA expression of TNF-α gene were detected at 4 h PI above the levels at 24 h PI in all groups.

**Figure 8:**
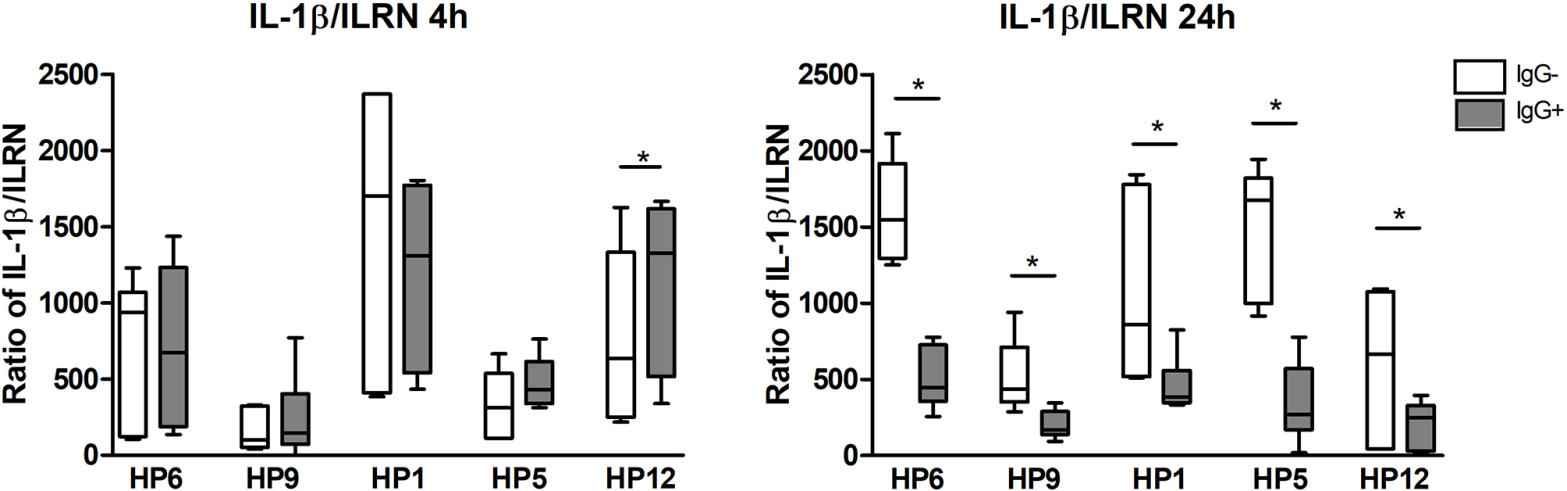
The ratio of mRNA expressions of IL-1β (pro-inflammatory cytokine) and IL-1Ra (the interleukin1-receptor antagonist, anti-inflammatory cytokine) at 4 and 24 h PI. Significant difference between groups with (IgG+) or without (IgG-) the post-vaccinal IgG is denoted by asterisk (p<0.05).

The ratio of mRNA expressions of IL-1β gene as the pro-inflammatory cytokine and IL-1RN gene (the interleukin1-receptor antagonist) as the anti-inflammatory cytokine is illustrated in the **Figure 8**. At 24 h this ratio is lower for every bacterial strain in the presence of IgG, which infers that the IgG antibodies elevated mRNA expression of anti-inflammatory IL-1RN gene at this post-infection time. At 4 h PI, there is no difference in the opsonized and non-opsonized bacteria, except for serovar 12.

**Figure 9:**
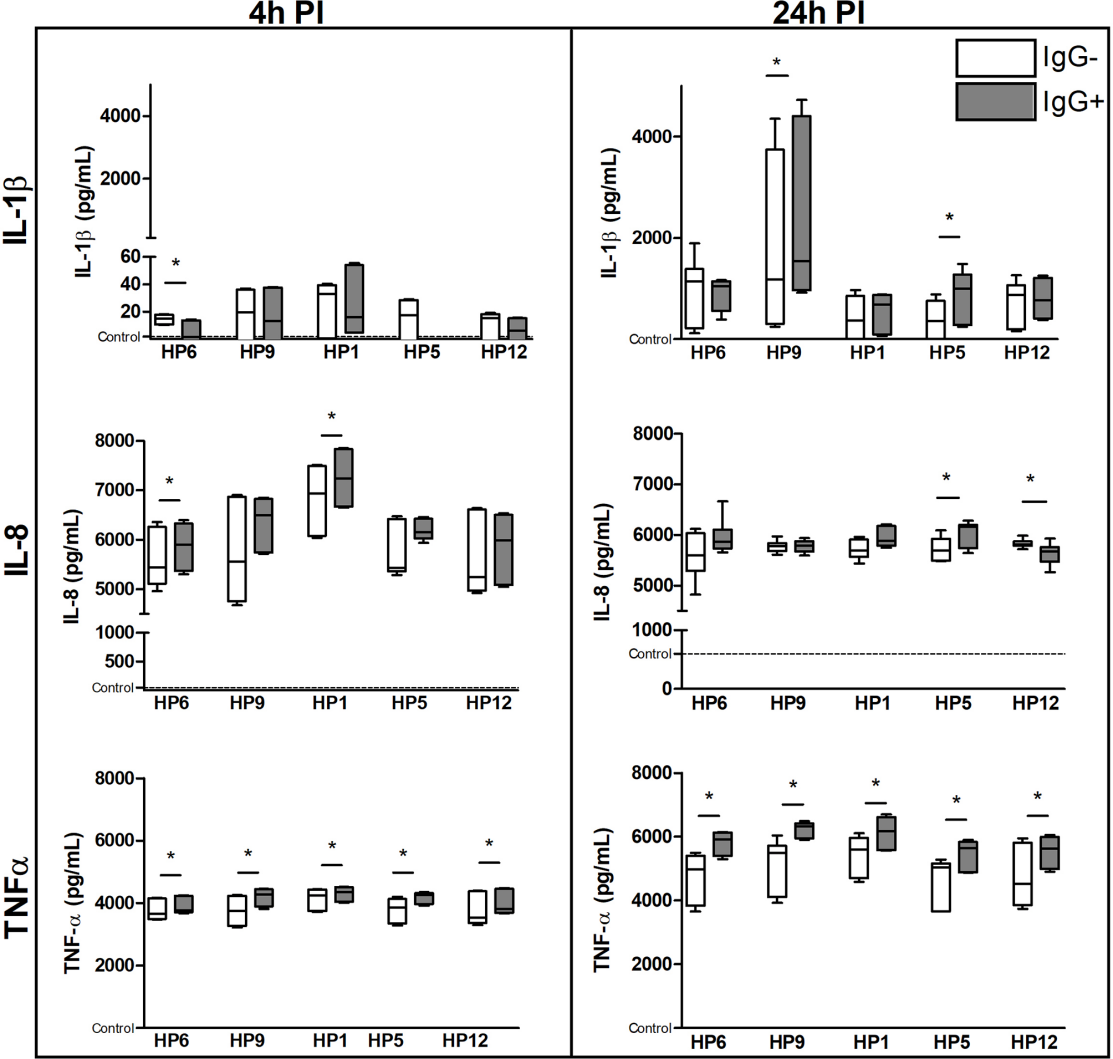
The immune response of PAMs at a protein level. PAMs were infected with various *G. parasuis* strains for 4 and 24 h. The concentration of IL-1β, IL-8 and TNF-α was measured by commercial ELISA kits at these PI times. Significant difference between groups with (IgG+) or without (IgG-) the post-vaccinal IgG is denoted by asterisk (p<0.05).Dotted line displays expression of mRNA in the control non-infected cells.

Concentrations of the selected cytokines at the protein level in the cell culture supernatants are shown in the **Figure 9**. Concentrations of IL-1RN gene product (i.e.IL-1Ra protein) and IL-10 were under the detectable levels of the used ELISA kits (data not shown). Production of IL-1β was lower after 4 h of infection than after 24 h and was significantly higher in PAMs infected with serovar 9 or 5 in the presence of IgG than without IgG at 24 h PI. Cells affected with serovar 1 or 6 for 4 h and with serovar 5 for 24 h produced significantly higher amount of IL-8 in the presence of IgG than without them. TNF-α was produced in much higher concentrations by PAMs affected with every strain that was pre-incubated with the IgG than without antibodies at both PI times.

## Discussion

*G. parasuis* is a commensal organism of the upper respiratory tract of pigs but it is also able to overcome the host innate immune system in the lungs (e.g. the alveolar macrophages) and cause systemic disease. The capsule is one of the virulence factors of this bacterium (4) and likely helps the bacterium to resist phagocytosis by alveolar macrophages (7). In our study, PAMs were able to recognize and phagocyte *G. parasuis* with strain-to-strain variability despite presence of the capsule observed in the virulent as well as in the avirulent strains. Differences in phagocytosis and in presence of the capsule of *G. parasuis* strains between our study and studies of other authors may be caused by different capsule structure, various gene expression in the main capsule loci under diverse conditions of growth or host environment, phagocytosis resistance mechanism or by other virulence factors (7–10, 24, 25). To determine whether IgG antibodies against the crude capsular extract of the virulent *G. parasuis* strain CAPM4675 increase phagocytosis of various *G. parasuis* strains *in vitro*, we isolated IgG from sera of the cCE-immunized pigs, incubated different *G. parasuis* strains with these IgG and subsequently infected PAMs. We used these antibodies because mice immunized with the cCE in our previous study were partially protected against the challenge with various *G. parasuis* strains and the anti-cCE antibodies in mice sera were able to bind to various strains in the Western-blotting (13). In addition, we proved the specific reactivity of the post-vaccinal antibodies from positive pig sera with the capsular extract as well as with various *G. parasuis* strains also by the Western-blotting, TEM and immunoprecipitation method.

We found that opsonization of bacteria by the post-vaccinal IgG led to enhanced phagocytosis of avirulent serovar 9 and virulent serovar 5 and 12 in the first two hours of infection. After 5 h of infection, previous opsonization had no effect on uptake of bacteria by macrophages. Moreover, opsonization of bacteria had no effect on their survival inside PAMs. Internalized bacteria were killed in 24 h after their phagocytosis.

Using mass spectrometry, we found some virulence-associated proteins in the capsular extract that reacted with the post-vaccinal IgG antibodies and thus may be also responsible for enhanced phagocytosis of *G. parasuis*. For example, we detected the virulence-associated trimeric autotransporters (VtaA) VtaA6 that reacted with positive pig sera and not with negative sera. An IgG antibody response against VtaA6 (beside VtaA1, 5, 8, 9 and 10) was also detected in sera of pigs after experimental challenge with *G. parasuis* Nagasaki strain but not after immunization with *G. parasuis* Nagasaki bacterin (26). Authors indicated only *in vivo* expression of these proteins. However, we were able to isolate the cCE with VtaA *in vitro*, likely because of the preparation method, and this extract was able to enhance antibody response in pigs. Two other virulence-associated trimeric autotransporters, VtaA8 and VtaA9, were revealed as surface proteins of virulent strain PC4-6P (serovar 12) that delayed phagocytosis of this strain by PAMs. In addition, when this strain was opsonized with monoclonal antibodies against these two VtaA its phagocytosis was enhanced (9). Although we did not detect these VtaA amongst the precipitated proteins, we observed the same phenomenon of delayed phagocytosis in serovar 5, 9 and 12 in comparison to the non-opsonized bacteria. Thus, we speculate that the post-vaccinal IgG antibodies promoted phagocytosis of these strains by binding to their VtaA6 or other VtaA detected in the cCE. On the other hand, the VtaA 8 and 9 were not detected in an avirulent strain F9 of serovar 6 that was properly internalized and degraded in acidic compartments of an early endosome (9). Therefore, the absence of VtaA in serovar 6 and 1 may be an explanation of no elevation in phagocytosis after their opsonization with the post-vaccinal antibodies in our study. Of course, other mechanisms or virulence factors may be involved in phagocytosis resistance.

Catalase and superoxide dismutase (SOD) were other detected virulence-associated proteins that precipitated with the post-vaccinal antibodies. These proteins are the antioxidants that bacteria use to neutralize destructive effects of reactive oxygen species (e.g., superoxide anion, hydrogen peroxide, singlet oxygen, hydroxyl radicals) produced during the oxidative burst (27). The luminol-amplified chemiluminescence is able to detect superoxide anion as well as hydrogen peroxide in the presence of peroxidases (28). Because decomposition of these reactive oxygen species is catalyzed by the superoxide dismutase and catalase, these two enzymes apparently play an important role in the oxidative stress resistance of *G. parasuis* observed in our study as well as by other authors (20, 29, 30). Moreover, catalase was identified among the secreted proteins of *G. parasuis* Nagasaki strain as immunoreactive with *G. parasuis* anti-Nagasaki convalescent sera (31). In our study, all *G. parasuis* strains opsonized with the post-vaccinal IgG significantly increased the CL signal in PAMs suggesting the inhibiting effect of these antibodies on the antioxidant mechanism of the bacteria. Neutralization of the antioxidant mechanisms of *G. parasuis* may lead to attenuation of this bacterium with subsequent lower ability to induce disease. Additionally, protective effect of antibodies against superoxide dismutase was observed in mice after *G. parasuis* lethal challenge (32). When we compared the translated amino acid sequences from whole-genome sequencing from the publication of Brockmeier et al. (33), protein sequences of catalase and SOD are very similar among serovars – approx. 99% identity among tested serovars for catalase and 94% for superoxide dismutase (data not shown). We may thus expect the cross-reactivity of the anti-cCE antibodies at some reasonable level.

In macrophages, ROS also mediate signal transduction through oxidation of susceptible cysteine residues present in a number of proteins that regulate NF-κB activation (34). *G. parasuis* activates p38 and the c-Jun amino-terminal kinase (JNK) that along with NF-κB activation lead to up-regulation of pro-inflammatory cytokines and chemokines in host cells (35–37). Our results of higher mRNA expression of pro-inflammatory TNF-α gene at 4 h PI and IL-1 β gene at both PI times indicate the stronger early immune response of macrophages in the presence of the post-vaccinal IgG opsonizing *G. parasuis*. Based on the results of ratio IL-1 β and IL-1RN gene expression, the early immune response may be substituted in presence of the post-vaccinal antibodies by higher expression of antagonist for IL-1β receptor at 24 h PI that may inhibit the negative effects of produced pro-inflammatory IL-1β to the surrounding tissues in host. The kinetics of their activation confirms the fact that IL-1Ra (i.e. IL-1RN gene product) is an important negative feedback regulator of IL-1β (38, 39). Additionally, these two cytokines may be influenced by another cytokine such as the anti-inflammatory cytokine IL-10 that down-regulates the expression of IL-1β and up-regulates the expression of IL-1RN genes (40). Higher expression of IL-10 gene detected in the infected PAMs at 24 h PI may be due to the potent effect of IL-1β and TNF-α produced in higher levels at this PI time (41, 42). Moreover, IL-10 inhibits the activity of TNF-α (43) that maybe a reason of TNF-α down-regulation observed at mRNA level at 24 h PI. TNF-α and IL-10 may play a role in the adaptive response of host organism to *G. parasuis* infection (44). Higher production of TNF-α by PAMs infected with *G. parasuis* pre-incubated with the post-vaccinal IgG than without the antibodies may indicate a role of this cytokine in a humoral response (44, 45). TNF-α is able to induce IL-8 production (43). IL-8 is a chemokine that attracts neutrophils to the inflammation site with their subsequent activation and ROS production leading to enhanced killing of pathogens (46). The mRNA expression of IL-8 gene was even higher in the presence of the post-vaccinal IgG than without IgG. Higher production of pro-inflammatory cytokines in the presence of IgG antibodies confirm the pro-inflammatory response following Fc-mediated phagocytosis (47, 48).

To summarize, opsonization of *G. parasuis* by antibodies against the crude capsular extract of strain CAPM 6475 enhanced phagocytosis of various strains independent of virulence. Different susceptibility of encapsulated *G. parasuis* strains to phagocytosis observed in our study could be due to different structure of the capsule, diverse gene expression for capsule production, or presence of various virulence factors like the VtaA. Moreover, opsonization of bacteria promoted phagocytosis up to 2 h PI which indicate the role of antibodies in the first hours of infection. Although *G. parasuis* infection of PAMs led to inhibition of ROS production (likely of superoxide anion and hydrogen peroxide), opsonization by the post-vaccinal IgG increased the oxidative burst. Because catalase and SOD were detected in the capsular extract and reacted with sera of the cCE-immunized pigs, we presume that these antibodies had neutralizing effects on catalase and SOD of *G. parasuis* in PAMs infection. Together with higher expression/production of pro- and anti-inflammatory cytokines, antibodies against the cCE may lead to attenuation of this bacterium with subsequent lower ability to induce disease at early stage of infection. Taken together, the crude capsular extract is a promising vaccine candidate. Therefore, our next step is to perform an experimental challenge of pigs with different *G. parasuis* strains after their immunization with the cCE vaccine.

## Data Availability Statement

The datasets presented in this study can be found in online repositories. The names of the repository/repositories and accession number(s) can be found in the article/**Supplementary Material**.

## Ethics Statement

The animal study was reviewed and approved by the Branch Commission for Animal Welfare of the Ministry of Agriculture of the Czech Republic (approval protocols No. MZe 1704 and MZe 1487).

## Author Contributions

KM and MF designed the study. KM prepared the crude capsular polysaccharide extract. KN immunized pigs. JG performed the mass spectrometry analysis and immunoprecipitation. PK performed the TEM and immunoelectron microscopy. RT performed the Western-blotting analysis. HK performed the ELISA test. KM and LK performed *in vitro* experiments. KM analyzed data, performed statistical analysis and wrote the manuscript. All authors contributed to the article and approved the submitted version.

## Funding

The study was supported by the project IGA VFU Brno 123/2017/FVL of the University of Veterinary and Pharmaceutical Sciences, the projects of the Ministry of Education, Youth and Sports of the Czech Republic (CZ.02.1.01/0.0/0.0/15_003/0000495 and CZ.1.05/2.1.00/19.0385) and the project RO 0518 of the Ministry of Agriculture of the Czech Republic.

## Conflict of Interest

The authors declare that the research was conducted in the absence of any commercial or financial relationships that could be construed as a potential conflict of interest.

## Supplementary Material

Supplementary Material 1 Proteins identified by mass spectrometry analysis of the crude capsular extract.
